# *Enterococcus faecium* E297 in the Diet of Chickens: Effects on Zootechnical Performance, Animal Health and Meat Quality

**DOI:** 10.1021/acsomega.6c02805

**Published:** 2026-10-07

**Authors:** Mariane Ficagna Bertotti, Maiara Marchiori, Paulo Vinicius de Oliveira, Alexandra Melissa Goncalves de Carvalho, Stéfani Mallmann, Tais Nunzio Muller, Gabriel Danieli, Júnior Mendes Furlan, Roger Wagner, Ricardo Evandro Mendes, Marcel Manente Boiago, Louise Haubert, Aleksandro Schafer Da Silva, Liziane Schittler Moroni

**Affiliations:** † Departamento de Engenharia de Alimentos e Engenharia Química, 74382 Universidade Do Estado de Santa Catarina (UDESC), Rodovia BR-282, Km 573.5, Linha Santa Terezinha, 89870-000 Pinhalzinho, SC, Brazil; ‡ Departamento de Zootecnia, 74382 Universidade Do Estado de Santa Catarina (UDESC), Rua Beloni Trombeta Zanin, 680-E, Santo Antônio, 89815-630 Chapecó, SC, Brazil; § Chromatography and Food Analysis Research Group, Federal University of Pampa (UNIPAM), Rua Luiz Joaquim de Sá Britto, S/n, Promorar, 97650-000 Itaqui, RS, Brazil; ∥ Departamento de Ciência e Tecnologia Dos Alimentos, 28118 Universidade Federal de Santa Maria (UFSM), Avenida Roraima, 1000, Camobi, 97105-900 Santa Maria, RS, Brazil; ⊥ Instituto Federal Catarinense (IFC), Campus Concórdia, Rodovia SC-283, S/n, Fragosos, 89703-720 Concórdia, SC, Brazil

## Abstract

This study evaluated the potential of *Enterococcus faecium* E297 as a functional dietary supplement for broiler chickens, assessing its effects on zootechnical performance, immune response, and meat quality. A total of 224 chicks were allocated to four treatments: a control diet, a diet containing an antibiotic growth promoter, a diet supplemented with *E. faecium* E297, and a commercial probiotic diet. Probiotic supplementation improved feed conversion and weight gain and increased leukocyte counts compared with the control group. Birds fed *E. faecium* E297 showed a higher villus-to-crypt ratio, suggesting greater nutrient absorption efficiency. Meat from probiotic-supplemented groups exhibited lower fat content and higher protein levels. No significant differences (*P* > 0.05) were observed in microbial counts of the litter, cloaca, or feces throughout the experimental period. The results indicate that *E. faecium* E297 has potential as an effective probiotic alternative to antibiotic growth promoters in poultry production.

## Graphical abstract

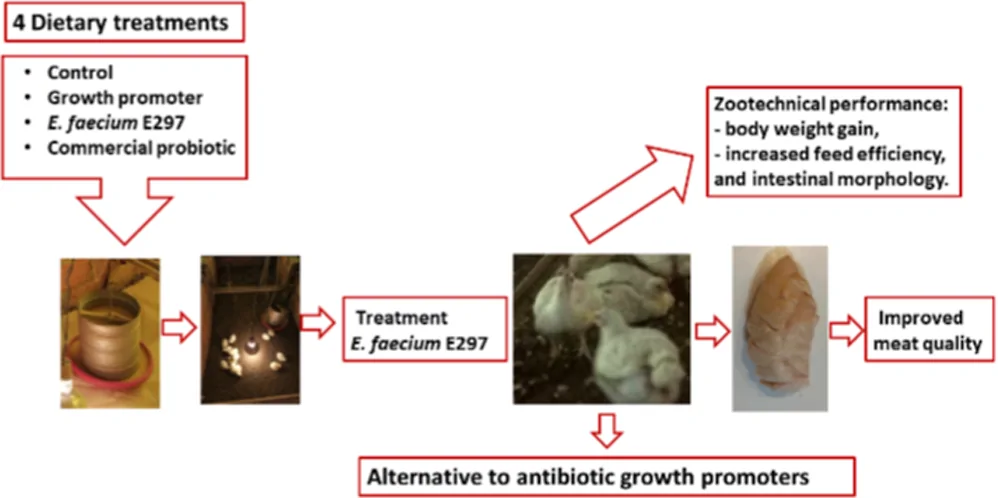

## 1 Introduction

Poultry farming is the fastest-growing sector among the main sources of animal protein worldwide; however, it still faces several challenges related to productivity and animal health.^1^ To meet the increasing global demand, multiple biosecurity and nutritional strategies are required to improve performance and ensure sustainable production.^2,3^ Poultry health and productivity are continuously threatened by various pathogens that can lead to economic losses due to increased mortality and morbidity, reduced feed conversion efficiency, and higher veterinary costs.^2,4^

The excessive use of antibiotics as growth promoters and therapeutic agents has raised growing public health concerns due to the emergence of antibiotic-resistant bacteria.^1^ Consequently, the poultry industry has intensified researches on natural alternatives to antibiotics, including probiotics, prebiotics, and other functional feed additives.

Probiotics are live microorganisms that, when administered in adequate amounts, confer health benefits to the host.^5^ Their mechanisms of action include inhibiting pathogen adhesion to intestinal epithelial cells, producing antimicrobial compounds such as bacteriocins and defensins, enhancing intestinal barrier integrity, lowering luminal pH, and modulating the immune system.^6–9^ Lactic acid bacteria (LAB) are among the most extensively used probiotics and are commonly found in the gastrointestinal tract of animals and in fermented foods.^10^ *Enterococcus faecium* was the first LAB species used as a probiotic food additive, having been approved by both the European Union and the Food and Drug Administration (FDA).^11^ Further, several studies have demonstrated that dietary supplementation with *E. faecium* can improve intestinal immunity, enhance mucin secretion in the jejunum, and promote growth performance and feed efficiency in broiler chickens, representing a safe and sustainable alternative to antibiotic growth promoters.^12–14^

The identification of new bacterial strains with probiotic potential is of great scientific and technological relevance, as probiotic activity is strain-dependent. Therefore, exploring bacterial diversity enables the selection of specific isolates with properties capable of benefiting the host. *E. faecium* E297 strain, the focus of this study, was isolated from raw milk in western Santa Catarina, Brazil. It produces antimicrobial substances, survives in acidic and neutral pH conditions (2–8), exhibits antagonistic activity, and harbors genes encoding enterocin production.^15^ When incorporated into a food matrix, this strain demonstrates enhanced resistance to gastrointestinal transit *in vitro*, suggesting its suitability as a probiotic candidate.^16^

Therefore, this study aimed to evaluate the effect of dietary inclusion of *E. faecium* E297 on the zootechnical performance, immune response (leukocyte counts), and meat quality of broiler chickens, and to verify its potential as a functional and sustainable alternative to conventional growth promoters.

## 2 Material and Methods

### 2.1 Materials

*E. faecium* E297 used in this study was characterized as probiotic,^15^ and belongs to the bacterial culture collection of the Food Microbiology Laboratory (Universidade do Estado de Santa Catarina, Pinhalzinho, SC, Brazil). The nucleotide sequence of 16S rRNA gene of probiotic strain was submitted in database of GenBank (Accession number OQ694960). The commercial probiotic *E. faecium* SF68 (Cylactin, DSM Nutrition and Animal Health, Kaiseraugst, Aargau, Switzerland) was used for comparison. The growth promoter Enradin F80 (Merck Animal Health, Rahway, USA) containing enramycin was used for the antibiotic treatment.

### 2.2 Animals and Experimental Design

A total of 224 one-day-old male broiler chicks (Cobb 500) were obtained from a local commercial hatchery (Chapecó, SC, Brazil). The study was carried out in an experimental aviary with the approval of the Ethics Committee for the Use of Animals at the Universidade do Estado de Santa Catarina, CEUA n° 1450170920. The birds were weighed and randomly distributed into 16 pens, being subjected to four experimental diets (treatments), with four replicates per treatment and 14 birds per replicate. The treatments were as follows: a control diet (C) consisting of a corn–soybean basal diet (BD); a growth promoter diet (GP) containing the BD supplemented with 10.0 mg/kg of enramycin; a probiotic diet (PE), containing the BD with 9.0 log CFU/g of *E. faecium* E297; and a commercial probiotic diet (CP), containing the BD with 9.3 log CFU/g of probiotic strain SF68 of diet. All groups received the same basal diet, provided through tubular feeders, differing only in the presence or absence of antibiotics or probiotics. The additives were incorporated into the basal diet during feed production at an experimental feed mill using a horizontal mixer, ensuring their homogeneous distribution in the diets provided to the birds. The experimental period lasted 41 days.

### 2.3 Preparation of the Basal Diet and Treatment Diets

The basal diet is detailed in Table S1 in the Supporting Information. *E. faecium* E297 was cultured in MRS broth at 36 °C for 18 h, centrifuged at 4000 rpm for 10 min (Centribio, Campinas, SP, Brazil), and washed with sterile distilled water. The resulting cells were resuspended in sterile distilled water, and 2.5 mL of this suspension was sprayed onto 1 kg of ground corn, followed by homogenization with the remaining feed ingredients using a mixer (Perozin, Concórdia, SC, Brazil), yielding a final concentration of 9.0 log CFU/g. For the preparation of the diet with the commercial probiotic *E. faecium* SF68, 15 mg/kg (9.3 log CFU/g) was added. All diet formulations were stored at room temperature.

### 2.4 Analyses

#### 2.4.1 Zootechnical Performance

The chickens and the remaining food in the buckets were weighed on days 1, 7, 21, 35, and 41 of the experiment to allow the calculation of weight gain (WG), feed intake (FI) (g/bird/day), and food conversion ratio (FCR). FI was calculated using the difference between the amount of food provided at the beginning and the weight of the leftovers at the end of each period. Feed conversion ratio (FCR) was calculated by dividing the total amount of food consumed by the live weight of the chickens.

#### 2.4.2 Sample Collection

On day 41 of the experiment, blood samples were collected from six broilers per treatment for biochemical and hematological analyses. Blood samples were collected by puncture of the ulnar vein using a 5 mL syringe fitted with a 25 × 7 mm needle. Of the collected blood, 2 mL was transferred to tubes containing 10% EDTA anticoagulant for hematological analyses, which were performed within 2 h of collection, while the remaining 3 mL was placed into tubes without anticoagulant for serum separation and subsequent biochemical analyses. Samples were maintained in refrigerated thermal containers during collection and transport to the laboratory. For serum separation, coagulated blood samples were centrifuged at 870 × g for 10 min. The resulting serum was collected and stored at −20 °C until analysis.

One bird per replicate (four per treatment) was subsequently euthanized following the ethical standards for animal welfare.^17^ Intestinal fragments (jejunum) were excised for histological assessment, whereas pectoral muscle was examined to determine protein content, pH, and fatty acid composition.

##### 2.4.2.1 Leucogram

At the time of blood collection, blood smears were prepared and stained for total leukocyte counts using a light microscope at 1000× magnification.

##### 2.4.2.2 Serum Biochemistries

Serum triglyceride levels were measured using a semiautomatic biochemical analyzer (BioPlus, Barueri, SP, Brazil).

##### 2.4.2.3 Histomorphometry

Jejunal tissue samples were fixed in 10% formaldehyde. Villus height was measured as the distance from the villus tip to the upper part of the crypt, and crypt depth as the distance from the base to the upper part of the crypt.

##### 2.4.2.4 Total Lipids Content and Fatty Acid Profile

Total lipid content and fatty acid (FA) profiles were determined in chicken breast samples. Lipid extraction was performed according to the method of Bligh and Dyer,^18^ with modifications described in detail by Reis et al.^19^ Fatty acid methyl esters (FAME) were prepared by transesterification following the method of Hartman and Lago.^20^ Briefly, the solvent from the lipid extract (corresponding to approximately 20 mg of fat) was evaporated under vacuum at 40 °C until complete dryness. Subsequently, 1 mL of 0.4 M KOH was added, and the mixture was vortexed and heated in a boiling water bath for 10 min. Then, 3 mL of 1.0 M H_2_SO_4_ was added, followed by vortex mixing and a further 10 min incubation in a boiling water bath. After the addition of 2 mL of hexane, samples were centrifuged at 5000 rpm for 5 min. The hexane phase containing the FAME was collected and subjected to gas chromatographic analysis.

FAME analysis was performed using a model 3400CX gas chromatograph equipped with a flame ionization detector (FID) (Varian, Palo Alto, CA, USA) and an automated injection system (Varian 8200 Autosampler). One microliter of each sample was injected into a split/splitless injector operated in split mode (50:1) at 230 °C. Hydrogen was used as the carrier gas at a constant pressure of 15 psi and an initial flow rate of 2.17 mL/min. Separation was achieved using a DB-WAX capillary column (30 m × 0.25 mm × 0.25 μm; J&W, Folsom, CA, USA). The oven temperature program started at 60 °C and was held for 1 min, increased to 180 °C at 20 °C/min, then to 220 °C at 2 °C/min, and finally to 230 °C at 10 °C/min, where it was maintained for 10 min. The detector temperature was set at 230 °C.

FAME were identified by comparing retention times with those of a commercial standard mixture (FAME Mix-37, Sigma-Aldrich, St. Louis, MO, USA). Results were expressed as the percentage of the total chromatographic area using flame ionization detector correction factors and ester-to-fatty-acid conversion factors, as described by Visentainer.^21^

##### 2.4.2.5 Measurement of pH Value, Fat and Protein of Broiler Meat

The pH was determined in 10 g chicken breast samples was measured using a pH meter (mPA-210, MS Tecnopon, Piracicaba, SP, Brazil). Each sample was measured in duplicate, and mean values were used for statistical analysis. Fat and protein analyses were performed by an accredited laboratory (LANAL Physical-Chemical Laboratory of Food Analysis, FIESC–SENAI, Chapecó, SC, Brazil).

#### 2.4.3 Microbiological Counts

Diet samples were collected on days 0, 7, 14, 21, 28, 35, and 41 for lactic acid bacteria (LAB) enumeration. Additional samples from aviary litter were collected and subjected to LAB and Enterobacteriaceae counts at time zero (day of animal housing), and on days 21, 35, and 41. Moreover, cloacal samples from the chickens were collected using a swab moistened with 0.1% peptone water (HiMedia, Maharashtra, India) on days 7, 21, and 35, and subjected to counts of Enterobacteriaceae, LAB, aerobic mesophilic microorganisms, and *Escherichia coli*. At 41 days, cecum feces were collected to count Enterobacteriaceae, LAB, mesophiles and *E. coli*. The samples were diluted in peptone water and inoculated on the surface of MRS Agar (Oxoid Ltd., Basingstoke, England), Plate Count Agar (PCA) (HiMedia, Maharashtra, India), Violet Red Bile Agar (VRB) (HiMedia, Maharashtra, India), and Eosin Methylene Blue Agar (EMB) (HiMedia, Maharashtra, India) for counting LAB, aerobic mesophilic microorganisms, Enterobacteriaceae, and *E. coli*, respectively. The plates were incubated at 37 °C for 24 h–48 h. Counts were performed and the results were expressed in log CFU/mL or g.

### 2.5 Statistical Analysis

Data were initially examined descriptively to ensure consistency and check assumptions. Results were expressed as mean ± standard deviation (SD). For each treatment and sampling time, two independent biological replicates were prepared and analyzed separately (n = 2). Normality was assessed using the Shapiro–Wilk test, and homogeneity of variance using Levene’s test. Variables related to zootechnical performance, as well as leukogram parameters, triglyceride levels, intestinal villus height, and the pH, fat percentage, and protein content of chicken meat, were analyzed using one-way ANOVA, followed by Tukey’s post hoc test when applicable. For microbial counts, which included repeated measurements over time, a repeated-measures ANOVA was applied to account for the temporal dependence among observations. Differences were considered statistically significant at *P* < 0.05. All statistical analyses were performed using R (v2.15.1).

## 3 Results

### 3.1 Zootechnical Performance

Performance results are summarized in Table 1. During the first week (days 1–7), broilers fed diets containing probiotics showed higher feed intake than those receiving enramycin (*P* < 0.001). However, no significant differences were observed in weight gain or feed conversion ratio among treatments during this period. From day 1 to day 41, broilers food diets supplemented with probiotics *E. faecium* E297 (PE) and SF68 (CP) achieved significantly greater weight gain compared with those on the basal diet (C) and the growth promoter diet (GP) (*P* < 0.001).

**Table 1:** Growth Performance Parameters of Broiler Chickens during the Starter, Grower, and Finisher Phases^a^ ^,^ ^b^

treatment	body weight (g)	feed intake (g)	feed conversion ratio
Starter Period (Day 1 to 7)			
C	147.70 (5.70)^a^	134.60 (8.40)^ab^	1.29 (0.13)^a^
GP	146.60 (7.30)^a^	126.70 (9.00)^b^	1.22 (0.04)^a^
PE	157.60 (11.70)^a^	139.90 (5.20)^a^	1.22 (0.05)^a^
CP	156.90 (8.10)^a^	140.20 (4.40)^a^	1.24 (0.06)^a^
*P* value	0.193	0.046	0.297
Grower Period (Day 1 to 21)			
C	868.90 (35.10)^b^	1138.50 (45.20)^b^	1.38 (0.06)^a^
GP	873.10 (39.40)^b^	1116.10 (45.10)^b^	1.34 (0.01)^ab^
PE	912.50 (41.40)^ab^	1193.70 (40.90)^ab^	1.37 (0.06)^a^
CP	950.00 (40.50)^a^	1203.50 (48.20)^a^	1.32 (0.02)^b^
*P* value	0.0012	0.0015	0.028
Finisher Period (Day 1 to 35)			
C	2411.10 (45.20)^b^	3587.00 (71.00)^ab^	1.48 (0.01)^a^
GP	2463.60 (42.20)^ab^	3453.00 (89.00)^b^	1.42 (0.01)^b^
PE	2435.20 (98.50)^ab^	3595.00 (48.00)^ab^	1.47 (0.04)^ab^
CP	2558.90 (37.40)^a^	3625.00 (60.00)^a^	1.44 (0.02)^b^
*P* value	0.0009	0.0018	0.016
Overall (Day 1 to 41)			
C	3036.00 (48.00)^c^	4858.00 (125.00)^a^	1.57 (0.02)^a^
GP	3167.00 (52.00)^bc^	4830.00 (111.00)^a^	1.52 (0.03)^b^
PE	3215.00 (98.00)^ab^	4890.00 (80.00)^a^	1.54 (0.04)^ab^
CP	3279.00 (101.00)^a^	4863.00 (14.00)^a^	1.53 (0.01)^b^
*P* value	0.0005	0.5983	0.0010

a Treatments consisted of a control diet (C; corn–soybean basal diet, BD), a growth promoter diet (GP; BD supplemented with 10.0 mg/kg enramycin), a probiotic diet (PE; BD supplemented with 9.0 log CFU/g of *E. faecium* E297), and a commercial probiotic diet (CP; BD supplemented with 9.3 log CFU/g of the probiotic strain SF68).

b Different letters on the same column indicate significant differences between groups using the Tukey test. Note: initial weight was 42,6 g (C), 42,8 g (GP), 43,3 g (PE) e 43,1 g (CP).

### 3.2 Sample Collection

#### 3.2.1 Leukogram

Leukogram data are shown in Table 2. Total leukocyte counts were significantly higher (5.68 ± 1.52 × 10^3^/μL) in broilers receiving the GP diet compared with those supplemented with probiotics (3.93 ± 1.14 × 10^3^/μL for PE and 3.33 ± 1.07 × 10^3^/μL for CP).

**Table 2:** Leukogram and Triglycerides of Broilers Fed with a Control Diet (C; corn–soybean Basal Diet, BD), a Growth Promoter Diet (GP; BD Supplemented with 10.0 mg/kg Enramycin), a Probiotic Diet (PE; BD Supplemented with 9.0 Log CFU/g of *E. faecium* E297), or a Commercial Probiotic Diet (CP; BD Supplemented with 9.3 Log CFU/g of the Probiotic Strain SF68)^a^

treatment	leukocytes (×10^3^/μL)	triglycerides (mg/dL)
C	2.18 (1.06)^b^	86.00 (19.0)^ab^
GP	5.68 (1.52)^a^	103.00 (29.0)^a^
PE	3.93 (1.14)^ab^	58.00 (24.0)^b^
CP	3.33 (1.07)^b^	85.00 (31.0)^ab^
*P* value	0.0002	0.0013

a Different letters on the same column indicate significant differences between groups using the Tukey test.

#### 3.2.2 Serum Biochemistry

Serum triglyceride levels (Table 2) were significantly higher (103 ± 29.0 mg/dL) in broilers receiving the GP diet compared with those supplemented with probiotics (58 ± 24.0 mg/dL) for PE and (85 ± 31.0 mg/dL) for CP.

#### 3.2.3 Histomorphometry

Intestinal villus height was greater in broilers fed the PE diet (982 ± 37 μm) than in those receiving the GP diet (952 ± 35 μm) (Table 3). The smallest crypt depth was observed in birds fed the basal diet (C) (214 ± 17 μm) and the PE diet (160 ± 11 μm) (*P* < 0.001), compared with the GP (216 ± 21 μm) and CP (219 ± 20 μm) groups. Consequently, the villus-to-crypt ratio was higher in the C (6.38) and PE (6.10) groups than in the GP (4.40) and CP (5.35) groups.

**Table 3:** Villus Height, Crypt Depth, and Villus-To-Crypt Ratio in the Jejunum of Broiler Chickens Fed a Control Diet (C; corn–soybean Basal Diet, BD), a Growth Promoter Diet (GP; BD Supplemented with 10.0 mg/kg Enramycin), a Probiotic Diet (PE; BD Supplemented with 9.0 Log CFU/g of *E. faecium* E297), or a Commercial Probiotic Diet (CP; BD Supplemented with 9.3 Log CFU/g of the Probiotic Strain SF68)^a^

treatment	villus (μm)	crypt (μm)	villus/crypt ratio
C	1368 (63)^a^	214 (17)^a^	6.38^a^
GP	952 (35)^b^	216 (21)^a^	4.41^c^
PE	982 (37)^b^	160 (11)^b^	6.10^a^
CP	1173 (54)^ab^	219 (20)^a^	5.35^b^
*P* value	0.004	0.0008	0.0012

a Different letters on the same column indicate significant differences between groups using the Tukey test.

#### 3.2.4 Fatty Acids in Animal Feed and Meat

No significant differences were found in total fat content among the diets (Table S2). However, the fatty acid composition of chicken meat varied significantly among treatments (Table S3). Meat from chickens fed with the GP diet had lower levels of total saturated fatty acids than chickens fed with the PE diet. Total monounsaturated fatty acids were lower in meat from chickens fed with probiotic diets (PE and CP) compared with those on the GP diet, whereas polyunsaturated fatty acids were higher in meat from the CP group than in the C and PE groups.

#### 3.2.5 Measurement of pH Value, Fat and Protein in the Broiler Meat

Meat pH values ranged from 5.86 to 5.98 across the treatments. Fat content varied from 13.5% to 19.9%, and protein content ranged from 14.3% to 22.2% (Table 4).

**Table 4:** Values of pH, Fat Percentage, and Protein in the Meat From Broiler Chickens Fed a Control Diet (C; corn–soybean Basal Diet, BD), a Growth Promoter Diet (GP; BD Supplemented with 10.0 mg/kg Enramycin), a Probiotic Diet (PE; BD Supplemented with 9.0 Log CFU/g of *E. faecium* E297), or a Commercial Probiotic Diet (CP; BD Supplemented with 9.3 Log CFU/g of the Probiotic Strain SF68)^a^

variable	C	GP	PE	CP	*P* value
pH	5.980^a^	5.860^b^	5.980^a^	5.970^a^	0.0006
fat (g/kg)	18.80^a^	19.900^a^	14.500^b^	13.500^b^	0.0011
protein (g/100 g)	21.730^a^	14.340^b^	20.470^a^	22.200^a^	0.0005

a *P* ≤ 0.05 indicates difference between treatments, different letters being used on the same line to show the difference between groups.

### 3.3 Microbiological Counts

#### 3.3.1 LAB Count in Broiler Feed

LAB counts in diets ranged from 2.10 ± 0.10 to 9.80 ± 0.20 log CFU/g during 41 days of storage at room temperature (Table 5). The lowest counts were observed in feed containing enramycin (C) on day 28, whereas the highest counts occurred in feed containing the commercial probiotic (PE) at day 35. Statistically, diets containing probiotics E297 (PE) and SF68 (CP) exhibited higher LAB counts than the control (C) and (GP) diets. LAB counts in the PE diet ranged from 7.30 ± 0.50 to 9.80 ± 0.20 log CFU/g, while in the CP diet they ranged from 7.05 ± 0.15 to 8.50 ± 0.50 log CFU/g.

**Table 5:** Lactic Acid Bacteria Count (LAB) (Log CFU/g) in the Diets Fed to Broiler Chickens During the 41 Day Experimental Period: a Control Diet (C; corn–soybean Basal Diet, BD), a Growth Promoter Diet (GP; BD Supplemented with 10.0 mg/kg Enramycin), a Probiotic Diet (PE; BD Supplemented with 9.0 Log CFU/g of *E. faecium* E297), and a Commercial Probiotic Diet (CP; BD Supplemented with 9.3 Log CFU/g of the Probiotic Strain SF68)^a^

	LAB (log CFU/g)						
treatment	day 0	day 7	day 14	day 21	day 28	day 35	day 41
C	5.64 (0.10)^bA^	4.15 (0.15)^bB^	3.80 (0.3)^bBC^	3.10 (0.10)^cD^	2.10 (0.10)^cD^	3.70 (0.10)^bBC^	2.60 (0.10)^cCD^
GP	4.27 (0.20)^bAB^	3.43 (0.13)^bBD^	4.30 (0.30)^bAB^	4.75 (0.15)^bA^	4.40 (0.2)^bAB^	3.80 (0.20)^bAB^	3.25 (0.15)^bB^
PE	9.29 (0.35)^aA^	8.89 (0.20)^aA^	7.30 (0.50)^aB^	8.80 (0.60)^aA^	8.80 (0.20)^aB^	9.80 (0.20)^aA^	9.10 (0.50)^aA^
CP	8.27 (0.35)^aAB^	8.50 (0.50)^aAB^	7.34 (0.34)^aBC^	7.05 (0.15)^aC^	8.00 (0.20)^aAB^	8.10 (0.50)^aD^	6.30 (0.30)^aD^
*P* value	0.0002	0.0015	0.005	0.001	0.0007	0.0004	0.0019

a Values are expressed as mean ± standard deviation of two independent biological replicates (*n* = 2). Data were analyzed using repeated-measures ANOVA. Different lowercase letters within the same column indicate significant differences among treatments (*P* < 0.05), whereas different uppercase letters within the same row indicate significant differences throughout storage time (*P* < 0.05).

#### 3.3.2 LAB and Enterobacteriaceae Counts in Aviary Litter

LAB counts in aviary litter ranged from 7.23 ± 0.75 to 9.54 ± 0.34 log CFU/g (Table 6). A significant reduction occurred only in the litter of broilers receiving the C diet, with concentrations decreasing from 9.54 ± 0.34 to 7.23 ± 0.75 log CFU/g over 41 days.

**Table 6:** Count of Lactic Acid Bacteria (LAB) and Enterobacteriaceae in Broiler Litter From Chickens Fed a Control Diet (C; corn–soybean Basal Diet, BD), a Growth Promoter Diet (GP; BD Supplemented with 10.0 mg/kg Enramycin), a Probiotic Diet (PE; BD Supplemented with 9.0 Log CFU/g of *E. faecium* E297), or a Commercial Probiotic Diet (CP; BD Supplemented with 9.3 Log CFU/g of the Probiotic Strain SF68)^a^

treatment	day 0	day 21	day 35	day 41
LAB (log CFU/g)				
C	9.54 (0.34)	8.43 (0.55)	8.34 (0.20)	7.23 (0.75)
GP	8.47 (0.95)	7.77 (0.80)	7.67 (0.74)	7.85 (0.88)
PE	8.72 (0.90)	9.31 (0.47)	9.01 (0.49)	8.81 (0.13)
CP	8.38 (0.34)	8.02 (0.69)	8.48 (0.85)	8.77 (0.76)
*P* value	0.191	0.460	0.402	0.257
Enterobacteriaceae (log CFU/g)				
C	4.13 (0.10)^aB^	6.13 (0.18)^aA^	3.04 (0.37)^aC^	3.95 (0.24)^bBC^
GP	2.78 (0.25)^bB^	5.07 (0.10)^bA^	3.50 (0.59)^aAB^	4.44 (0.21)^abAB^
PE	2.74 (0.10)^bB^	4.86 (0.36)^bA^	3.24 (0.34)^aB^	4.69 (0.10)^aA^
CP	2.77 (0.10)^bC^	4.83 (0.13)^bA^	3.68 (0.40)^aBC^	4.08 (0.18)^abAB^
*P* value	0.0010	0.003	0.622	0.0012

a Values are expressed as mean ± standard deviation of two independent biological replicates (*n* = 2). Data were analyzed using repeated-measures ANOVA. Different lowercase letters within the same column indicate significant differences among treatments (*P* < 0.05), whereas different uppercase letters within the same row indicate significant differences throughout storage time (*P* < 0.05).

Enterobacteriaceae counts ranged from 2.74 ± 0.10 to 6.13 ± 0.18 log CFU/g. Enterobacteriaceae counts increased significantly between days 0 and 21 in all treatments. The C group increased from 4.13 ± 0.10 to 6.13 ± 0.18 log CFU/g, while the GP, PE, and CP groups increased from 2.78 ± 0.25 to 5.07 ± 0.10, 2.74 ± 0.10 to 4.86 ± 0.36, and 2.77 ± 0.10 to 4.83 ± 0.13 log CFU/g, respectively. On day 35, a reduction in counts was observed in all groups, with values of 3.04 ± 0.37­(C), 3.50 ± 0.59 (GP), 3.24 ± 0.34 (PE), and 3.68 ± 0.40 (CP) log CFU/g. At day 41, counts increased again, ranging from 3.95 ± 0.24 to 4.69 ± 0.10 log CFU/g; however, they remained lower than those observed on day 21.

#### 3.3.3 LAB and Enterobacteriaceae Counts in Cloaca

LAB counts in cloacal swabs ranged from 5.50 ± 0.71 to 7.89 ± 0.97 log CFU/mL. No significant differences (*P* > 0.05) were detected among diets at the same sampling times. However, in broilers fed the GP diet, LAB counts decreased significantly from day 7 (7.63 ± 0.50 log CFU/mL) to day 35 (5.92 ± 0.11 log CFU/mL) (Table 7). Enterobacteriaceae counts ranged from 6.14 ± 0.10 to 8.40 ± 0.10 log CFU/mL, with significant differences observed only on day 7, when broilers in the CP group exhibited higher concentrations (6.42 ± 0.20 log CFU/mL) compared with the C (6.18 ± 0.10 log CFU/mL) and PE (6.14 ± 0.10 log CFU/mL) groups.

**Table 7:** Counts of Lactic Acid Bacteria (LAB) and Enterobacteriaceae in Cloaca Samples From Chickens Fed a Control Diet (C; corn–soybean Basal Diet, BD), a Growth Promoter Diet (GP; BD Supplemented with 10.0 mg/kg Enramycin), a Probiotic Diet (PE; BD Supplemented with 9.0 Log CFU/g of *E. faecium* E297), or a Commercial Probiotic Diet (CP; BD Supplemented with 9.3 Log CFU/g of the Probiotic Strain SF68)^a^

treatment	day 7	day 21	day 35
LAB (log CFU/g)			
C	7.89 (0.97)^aA^	7.33 (0.48)^aA^	5.50 (0.71)^aA^
GP	7.63 (0.50)^aA^	6.85 (0.43)^aAB^	5.92 (0.11)^aB^
PE	6.84 (0.09)^aA^	7.31 (0.33)^aA^	6.72 (0.34)^aA^
CP	7.36 (0.28)^aA^	7.35 (0.66)^aA^	6.95 (2.08)^aA^
*P* value	0.498	0.753	0.453
Enterobacteriaceae (log CFU/g)			
C	6.18 (0.10)^bC^	7.37 (0.10)^aB^	8.40 (0.10)^aA^
GP	6.30 (0.20)^abB^	7.01 (0.47)^aB^	8.19 (0.10)^aA^
PE	6.14 (0.10)^bB^	7.35 (0.29)^aA^	8.38 (0.34)^aA^
CP	6.42 (0.20)^aC^	7.40 (0.15)^aB^	8.28 (0.10)^aA^
*P* value	0.003	0.616	0.612

a Values are expressed as mean ± standard deviation of two independent biological replicates (*n* = 2). Data were analyzed using repeated-measures ANOVA. Different lowercase letters within the same column indicate significant differences among treatments (*P* < 0.05), whereas different uppercase letters within the same row indicate significant differences throughout storage time (*P* < 0.05).

#### 3.3.4 Enterobacteriaceae, LAB, Mesophiles, and *E. coli* Counts in Feces

Enterobacteriaceae counts in faecal samples ranged from 7.08 ± 0.42 to 7.23 ± 0.80 log CFU/g across all treatments (C, GP, PE, and CP). LAB, mesophile, and *E. coli* counts ranged from 7.71 ± 0.47 to 8.02 ± 0.64, 7.18 ± 0.94 to 8.35 ± 0.64, and 7.13 ± 0.55 to 8.24 ± 0.53 log CFU/g, respectively (Table 8). No significant differences (*P* > 0.05) were observed in these microbiological parameters among the treatments after 41 days.

**Table 8:** Count of Enterobacteriaceae, Lactic Acid Bacteria (LAB), Mesophiles and *Escherichia coli* (Log CFU/g) in Feces of Broilers Fed a Control Diet (C; corn–soybean Basal Diet, BD), a Growth Promoter Diet (GP; BD Supplemented with 10.0 mg/kg Enramycin), a Probiotic Diet (PE; BD Supplemented with 9.0 Log CFU/g of *E. faecium* E297), or a Commercial Probiotic Diet (CP; BD Supplemented with 9.3 Log CFU/g of the Probiotic Strain SF68)^a^

	log CFU/g			
treatment	Enterobacteriaceae	LAB	mesophiles	*E. coli*
C	7.03 (0.65)	7.71 (0.47)	7.46 (0.87)	7.13 (0.55)
GP	7.23 (0.80)	7.84 (0.78)	8.35(0.64)	7.52 (0.88)
PE	7.08 (0.42)	8.02 (0.64)	7.18 (0.94)	7.37 (0.80)
CP	6.13 (0.61)	7.72 (0.51)	7.76 (0.61)	8.24 (0.53)
*P* value	0.443	0.517	0.230	0.161

a Values are expressed as mean ± standard deviation of two independent biological replicates (*n* = 2). Data were analyzed using repeated-measures ANOVA. No significant differences were observed among treatments (*P* > 0.05).

## 4 Discussion

Probiotics have been widely employed in poultry production as natural alternatives to antibiotic-based growth promoters. In the present study, broiler chickens fed diets supplemented with probiotics *E. faecium* E297­(PE) and SF68 (CP) exhibited superior zootechnical performance compared with those receiving the basal diet (C) or the diet supplemented with a growth promoter (GP).

Birds fed with the diet supplemented with *E. faecium* E297 (PE) showed improved feed conversion efficiency and higher weight gain. These effects may be associated with the positive influence of probiotics on nutrient utilization and intestinal health, as previously reported in the literature. Probiotic microorganisms have been described as capable of producing antimicrobial metabolites, promoting beneficial gut microbiota colonization, and modulating the immune system, which may contribute to improved bird performance.^6–9^ Similar findings have been reported in previous studies demonstrating improvements in growth performance and immune modulation in broiler chickens supplemented with probiotics.^22–24^

With regard to the hematological profile, all experimental groups presented values within the physiological limits for healthy chickens, which agrees with previous studies.^25,26^ However, the number of leukocytes was significantly influenced by diet. Birds fed with *E. faecium* E297 (PE) or the growth promoter (GP) diet showed higher leukocyte counts compared with the control and commercial probiotic (CP) groups. The higher leukocyte counts observed in the PE group relative to the CP group are particularly relevant, as they indicate the immunomodulatory potential of *E. faecium* E297. This is consistent with Obianwuna et al.,^27^ who reported that lymphocyte proliferation is affected by gut microbiota modulation, with probiotic activity serving as a marker of immune competence in broilers.

Triglyceride levels were significantly lower in the serum of chickens receiving *E. faecium* E297 (PE) compared with those receiving the growth promoter (GP), confirming its potential in the regulation of lipid metabolism. This observation agrees with Dev et al.^28^ and Younas et al.,^22^ who reported reduced triglyceride levels in broilers fed with probiotic-supplemented diets compared to antibiotic-treated birds. Zhang et al.^29^ also demonstrated that probiotics regulate lipid deposition and improve fatty acid composition through activation of the AMPK signaling pathway, corroborating the findings of the present study.

Probiotic supplementation also affected intestinal morphology. In this study, villus height increased and crypt depth decreased in broilers receiving *E. faecium* E297 (PE), resulting in an improved villus-to-crypt ratio compared with birds fed the growth promoter (GP) diet. Similar morphological improvements have been reported following probiotic administration in poultry.^22,30^ The increase in villus surface area enhances nutrient absorption, whereas the reduction in crypt depth indicates decreased epithelial cell turnover and lower energy expenditure for tissue maintenance.^22,23,31^

The fatty acid composition of meat is an important indicator of nutritional quality. In this study, chickens fed using probiotics (PE and CP) exhibited reduced saturated fatty acid levels and higher proportions of unsaturated fatty acids compared with other treatments. These findings are consistent with previous reports linking probiotic supplementation to improved lipid profiles and better health indices in poultry meat.^32,33^ Furthermore, broilers fed with probiotic diets (PE and CP) showed lower fat and higher protein content in the meat compared with those receiving the growth promoter (GP), corroborating to other studies associating probiotic inclusion with improved carcass quality.^28,34^

Meat pH is a key determinant of post-mortem biochemical processes, affecting texture, color, shelf life, and consumer acceptance.^35^ In this study, the lowest pH values were observed in chickens fed with the GP diet, while the highest were recorded in those fed with the PE diet. These results suggest that diet composition, particularly probiotic inclusion, can influence the biochemical conversion of muscle to meat, in line with the observations of Tang et al.^36^ and Zhang et al.^29^

The genus *Enterococcus* belongs to the lactic acid bacteria (LAB) group and is widely studied as a probiotic in poultry feeding.^22,27,30,35,37,38^ In the present study, LAB concentrations in feeds supplemented with probiotics (PE and CP) remained within the recommended threshold (≥10^6^ CFU/g) throughout 41 days of storage, confirming probiotic stability under standard feed conditions.^39^ The best viability results were observed for *E. faecium* E297, which outperformed the commercial probiotic strain SF68.

Despite the viability of the probiotics in feed, no significant differences were observed in the counts of LAB, mesophilic bacteria, *E. coli*, or Enterobacteriaceae in poultry litter, cloacal, or fecal samples at the end of the experiment. These findings could be by established microbiota in the poultry litter exerted a greater influence on the environmental microbial dynamics than probiotic supplementation itself. Also, future molecular analyses are needed to determine whether functional or compositional changes have occurred. Poultry litter harbors a complex microbial community predominantly composed of Gram-positive bacteria, including *Lactobacillus*, *Enterococcus*, *Pediococcus*, and *Bacillus*, which may promote ecological stability through competition for nutrients and adhesion sites, as well as through the production of antimicrobial metabolites that influence microbial dynamics within the environment.^40–42^ Although several studies have reported reductions in coliform populations following probiotic supplementation,^23,43^ others did not observe significant changes in microbial counts,^44,45^ which is consistent with the findings of the present study. In addition, studies performed by Lu et al.,^46^ Wang et al.,^47^ and Kubasova et al.^48^ demonstrated that probiotic supplementation does not always result in significant reductions of Enterobacteriaceae when the environmental microbiota is already established.

The absence of significant differences in microbial counts may also be associated with limitations related to the microbiological methods employed and the experimental sampling itself. Culture-based and plate-count methods evaluate only cultivable microorganisms and may therefore underestimate important changes in the intestinal and environmental microbiota. Moreover, the high variability of microbiota composition among birds and its dynamic nature throughout the rearing period may hinder the detection of subtle treatment-related differences.^49–51^

Therefore, the lack of significant differences in microbial counts does not necessarily indicate the absence of probiotic activity. Probiotics may exert beneficial effects primarily through functional mechanisms, including modulation of the intestinal microbiota, competitive exclusion of pathogens, enhancement of gut barrier integrity, and stimulation of the immune system, without necessarily causing marked changes in cultivable microbial populations.^52,53^ This hypothesis is supported by the results obtained in the present study, in which supplementation with *E. faecium* E297 improved zootechnical performance, body weight gain, feed efficiency, intestinal morphology, and the lipid profile of broiler meat. Collectively, these findings indicate that *E. faecium* E297 has strong potential as a functional probiotic culture for poultry production, representing a natural and sustainable alternative to antibiotic growth promoters.

### 4.1 Study Limitations and Future Perspectives

Despite the insights provided by this study, some limitations should be acknowledged. The relatively small sample size and the high variability observed in microbiological data may limit the generalization of the findings, suggesting that larger experimental groups are needed to confirm the observed trends. In addition, the use of culture-based microbiological methods may not fully capture the complexity of the intestinal microbiota when compared with culture-independent approaches, such as 16*S* rRNA gene sequencing and metagenomic or metabolomic analyses, which provide a more comprehensive characterization of microbial diversity and functional activity.^46,47^ Therefore, the absence of significant differences in microbial counts does not exclude functional modulation of the gut ecosystem associated with probiotic supplementation, since metabolic and compositional shifts may occur without changes in total cultivable bacterial populations.

## Supplementary Material



## Notes

Table S1: ingredients and calculated chemical composition of the experimental diets; Table S2: total lipid and fatty acid profile of the experimental diets; Table S3: fatty acid profile of broiler meat (PDF)
