# Supplementary material for: Enterococcus faecium E297 in the Diet of Chickens: Effects on Zootechnical Performance, Animal Health and Meat Quality
Source: ACS Omega. 2026 Oct 7;11(41):62572–80. doi: 10.1021/acsomega.6c02805 (PMC13689151; doi:10.1021/acsomega.6c02805)
Supplement: Supplementary file 1 [file ao6c02805_si_001.pdf]

Supporting Information

***Enterococcus faecium* E297 in the diet of chickens: effects on zootechnical performance, animal health and meat quality**

Mariane Ficagna Bertotti[1], Maiara Marchiori[2], Paulo Vinicius de Oliveira[2], Alexandra de Carvalho[1], Stéfani Mallmann[1], Tais Muller[1], Gabriel Danieli[1], Júnior Furlan[3], Roger Wagner[4], Ricardo Evandro Mendes[5], Marcel Manente Boiago[1, 2], Louise Haubert[1], Aleksandro Schafer Da Silva[2], Liziane Schittler Moroni[1*]

[1] Departamento de Engenharia de Alimentos e Engenharia Química, Universidade do Estado de Santa Catarina (UDESC), Rodovia BR-282, km 573,5, Linha Santa Terezinha, Pinhalzinho, SC, 89870-000, Brazil.

[2] Departamento de Zootecnia, Universidade do Estado de Santa Catarina (UDESC), Rua Beloni Trombeta Zanin, 680-E, Santo Antônio, Chapecó, SC, 89815-630, Brazil.

[3] Chromatography and Food Analysis Research Group, Federal University of Pampa (UNIPAM), Rua Luiz Joaquim de Sá Britto, s/n, Promorar, Itaqui, RS, 97650-000, Brazil

[4] Departamento de Ciência e Tecnologia dos Alimentos, Universidade Federal de Santa Maria (UFSM), Avenida Roraima, 1000, Camobi, Santa Maria, RS, 97105-900, Brazil.

[5] Instituto Federal Catarinense (IFC), Campus Concórdia, Rodovia SC-283, s/n, Fragosos, Concórdia, SC, 89703-720, Brazil.

[*] **Corresponding author:** Liziane Schittler Moroni - Departamento de Engenharia de Alimentos e Engenharia Química, Universidade do Estado de Santa Catarina (UDESC), Rodovia BR-282, km 573,5, Linha Santa Terezinha, Pinhalzinho, SC, 89870-000, Brazil. Email: liziane.schittler@udesc.br

Supplementary material

Table S1. Ingredients and basal diet used for all experimental groups

Ingredients (%)	Age (days)		
	1 – 21	22 - 35	36 – 45
Corn	55.15	58.00	62.10
Soybean meal	37.32	33.7	29.82
Soy oil	3.90	4.93	4.97
Bicalcium phosphate	1.27	1.30	1.12
Calcitic limestone	1.14	0.92	0.80
Iodized salt	0.48	0.43	0.43
DL-Methionine – 99%	0.29	0.28	0.25
L-lysine – 78%	0.20	0.19	0.26
L-threomine – 99%	0.05	0.05	0.05
Premix of vitamins and minerals[1]	0.20	0.20	0.20
Calculated chemical composition	100	100	100
Energy (kcal/kg)	3050	3150	3200
Brute protein (%)	21.20	19.80	18.40
Calcium (%)	0.84	0.76	0.66
Available phosphorus (%)	0.40	0.35	0.31
Digestible lysine (%)	1.22	1.13	1.06
Digestible methionine (%)	0.47	0.45	0.42
Digestible methionine + cysteine (%)	0.88	0.83	0.77
Digestible threonine (%)	0.79	0.73	0.69
Digestible tryptophan (%)	0.21	0.20	0.19
Sodium (%)	0.21	0.20	0.19

[1] Minimal vitamin and mineral levels per kg of product: vitamin A (5.000.000 UI); vitamin D3 (1.000.000 IU); vitamin E (15.000 UI); vitamin K3 (1.500 mg); vitamin B1 (1.500 mg); vitamin B2 (3.000 mg); vitamin B6 (2.000 mg); vitamin B12 (7.000 mcg); folic acid (500 mg); nicotinic acid (15 g); pantothenic acid (7000 mcg); choline (80 g); biotin (100 mg); Copper (10 g); iron (50 g); iodine (1.000 mg); manganese (80 g); selenium (300 mg); zinc (70 g); minimum humidity (20 g); maximum mineral matter (980 g).

Table S2. Total lipids and fatty acid profile in the feed of chickens fed with the following diets: C (corn–soybean basal diet — BD); GP (BD containing 10.0 mg/kg of enramycin); PE (BD containing 9 log CFU/g of probiotic E297); and CP (BD containing 9.3 log CFU/g of probiotic SF68)

Variables	C	GP	PE	CP
Total Fat	7,34	7,52	7,52	7,62
C14:0 - myristic acid	0,093	0,102	0,096	0,110
C16:0 - palmitic acid	16,38	16,29	16,42	15,93
C16:1n7 - palmitoleic acid	0,101	0,095	0,111	0,095
C17:0 - heptadecanoic acid	0,103	0,103	0,103	0,102
C18:0 - stearic acid	3,947	4,160	3,977	4,081
C18:1 cis (n9) - oleic acid	28,78	28,47	29,02	29,03
C18:2 cis (n6) - linoleic acid	46,12	46,16	45,78	46,13
C20:0 - arachidic acid	0,369	0,400	0,391	0,396
C18:3n3 - α-linolenic acid	3,848		3,747	3,772
C22:0 - behenic acid	0,352		0,338	0,343

NOTE: there was no statistical difference (P>0.05) between treatments for the variables present in the table.

Table S3. Fatty acid profile in meat of broiler chicken fed with the following diets: C (corn–soybean basal diet — BD); GP (BD containing 10.0 mg/kg of enramycin); PE (BD containing 9 log CFU/g of probiotic E297); and CP (BD containing 9.3 log CFU/g of probiotic SF68)

Variables (%)	C	GP	PE	CP	Value-P
C14:0 - myristic acid	0,624	0,594	0,572	0,569	0.589
C14:1n5 - myristoleic acid	0,073	0,072	0,046	0,054	0.061
C15:0 - pentadecanoic acid	0,103[bc]	0,121[a]	0,097[c]	0,107[ab]	0.024
NI	2,931[b]	3,038[b]	4,203[a]	4,073[a]	<0.001
C16:0 - palmitic acid	31,490[a]	28,720[b]	31,850[a]	31,250[a]	0.007
C16:1n9	0,382	0,415	0,402	0,394	0.726
C16:1n7 - palmitoleic acid	3,044	2,704	2,262	1,999	0.054
C17:0 - heptadecanoic acid	0,587	0,593	0,745	0,662	0.251
NI	0,134	0,129	0,122	0,137	0.840
C17:1n7 - cis-10-heptadecenoic acid	0,339	0,550	0,572	0,577	0.189
C18:0 - stearic acid	8,279	8,984	9,088	8,380	0.782
C18:1 trans isomer	0,082	0,105	0,086	0,079	0.628
C18:1 cis (n9) - oleic acid	24,710[a]	25,260[a]	22,770[b]	22,510[b]	0.003
C18:1 cis (n11) - vaccenic acid	1,777	1,785	1,817	1,605	0.872
C18:2 cis (n6) - linoleic acid	21,360	22,080	20,780	23,470	0.087
C18:3n6 - γ-linolenic acid	0,086[ab]	0,106[a]	0,049[b]	0[c]	<0.001
C20:0 - arachidic acid	0,038[a]	0,049[a]	0,021[b]	0[c]	<0.001
C18:3n3 - α-linolenic acid	1,179	1,322	1,104	1,223	0.364
C20:1(n9) - cis-11-eicosenoic acid	0,121	0,171	0,121	0,111	0.927
C20:2n6 - cis-11,14-eicosadienoic acid	0,217	0,347	0,311	0,285	0.098
C20:3n6 - cis-8,11,14-eicosatrienoic acid	0,278	0,368	0,365	0,303	0.549
C20:4n6 - arachidonic acid	1,758	1,952	2,130	1,802	0.180
C24:1n9 - nervonic acid	0,285[c]	0,408[a]	0,355[ab]	0,328[bc]	0.050
C22:6(n3) (DHA) - cis-4,7,10,13,16,19-docosahexaenoic acid	0,101[a]	0,106[a]	0,106[a]	0,056[b]	<0.001
Saturated fatty acids	41,120[ab]	39,060[b]	42,380[a]	40,970[ab]	0.035
Monounsaturated fatty acids	30,430[a]	31,060[a]	28,030[b]	27,27[b]	0.002
Polyunsaturated fatty acids	24,980[b]	26,280[ab]	24,850[b]	27,140[a]	<0.001

Note: P≤0.05 indicates difference between treatments, different letters being used on the same line to show the difference between groups.
